# Public attitudes toward the use of human induced pluripotent stem cells: insights from an Italian adult population

**DOI:** 10.3389/fpubh.2024.1491257

**Published:** 2024-11-06

**Authors:** Noemi Elia, Federica Prinelli, Valeria Peli, Silvia Conti, Mario Barilani, Cecilia Mei, Silvana Castaldi, Lorenza Lazzari

**Affiliations:** ^1^Unit of Cell and Gene Therapy, Fondazione IRCCS Ca' Granda Ospedale Maggiore Policlinico, Milan, Italy; ^2^Epidemiology and Public Health Unit, Institute of Biomedical Technologies - National Research Council, Segrate (MI), Italy; ^3^Dino Ferrari Center, Department of Pathophysiology and Transplantation, University of Milan, Milan, Italy; ^4^Quality Unit, Fondazione IRCCS Ca' Granda Ospedale Maggiore Policlinico, Milan, Italy; ^5^Department of Biomedical Sciences for Health, University of Milan, Milan, Italy

**Keywords:** human induced pluripotent stem cells, internet-based survey, public attitude, citizen participation, stem cell research

## Abstract

**Introduction:**

Human induced pluripotent stem cells (hiPSCs), derived from reprogrammed adult somatic cells, hold significant promise for disease modelling, personalized medicine, drug discovery, and regenerative therapies. Public awareness and understanding of hiPSCs are crucial for advancing research in this field. However, limited data exists on the general population’s knowledge and attitudes toward their use.

**Methods:**

This study aimed to assess the awareness and perceptions of hiPSCs among Italian adults through a web-based survey conducted via the EUSurvey platform, using a snowball sampling approach. The survey included demographic information and mandatory questions on knowledge, awareness, and concerns regarding hiPSC technology, with responses collected on a 3-point scale. Statistical analysis was performed using chi-squared tests, with significance set at *p* ≤ 0.05.

**Results:**

Out of 1874 respondents, the majority were aged 18–35 years (40.5%), female (63.4%), and university-educated (67.2%). Among those familiar with hiPSCs (54.1%, *n* = 1,201), 95.3% expressed willingness to donate blood samples for hiPSC generation to treat individuals with incurable diseases. Concerns about current research and therapeutic applications were low (less than 20%), but nearly half of the respondents were hesitant or opposed to the use of hiPSCs in animal experiments and their commercialization by pharmaceutical companies. Increased skepticism was observed in older, less educated, religious individuals, and those who were not blood donors. Overall, the Italian public shows strong support for hiPSC-based therapies, though reservations exist around specific ethical and economic issues.

**Discussion:**

These findings underscore the importance of addressing public concerns through targeted educational campaigns, not only in Italy but globally, to foster a more informed and supportive environment for advancing stem cell research and its clinical applications worldwide. Similar studies have been conducted in Japan, the United States, and Sweden, but there remains a need for all countries to engage with their citizens to better understand how stem cell research is perceived locally. Such engagement is crucial for guiding international strategies in personalized medicine and regenerative therapies, ensuring that emerging technologies are met with both ethical integrity and public trust.

## 1 Introduction

Human induced pluripotent stem cells (hiPSCs) are generated from adult somatic cells (e.g., skin fibroblasts or blood cells) by genetic reprogramming to an embryonic state. When the genes encoding specific transcription factors are introduced into adult cells, they acquire the key features of the stem status, i.e., the ability to differentiate into almost every cell type and to self-renew. hiPSCs were first reported in Japan in 2007 when Professor Yamanaka reprogrammed mouse fibroblasts (1), followed by human fibroblasts (2). Because of their pluripotent stem state, hiPSCs offer an unlimited supply of a broad variety of patient-specific cells for research and therapeutic applications.

The discovery of hiPSCs overcame the ethical concerns associated with human embryonic stem cells (hESCs), which can be derived from the inner cell mass of a 5-to 7-day-old blastocyst and whose production involves the destruction of human embryos (3, 4). hESCs are considered the gold standard of pluripotent stem cells, but their research is associated with many public and political controversies, as people have different scientific, religious, and moral beliefs; additionally, the utilization of these early-stage embryos has generated much debate and controversy among pro-life movement supporters and stem-cell research opponents (5).

In the field of stem cell research, at the beginning of the 21st century, human multipotent stem cells isolated from fetal and adult sources, such as amniotic fluid (6, 7) and cord blood (8–11), were proposed for research and clinical applications as they do not raise any particular ethical concerns. However, it is still controversial whether these cells can be expanded indefinitely and, most importantly, whether they are pluripotent, i.e., have the potential to differentiate into any of the three germ layers (12, 13).

hiPSCs combine the best properties of hESCs and adult stem cells, avoiding most ethical debates, since no embryos or oocytes are used to generate hiPSCs. Notably, they provide a virtually unlimited source of stem cells as they can be propagated indefinitely and have the intrinsic potential to differentiate into any cell type. For these reasons, hiPSCs hold great potential for disease modeling, personalized therapies, drug discovery, and regenerative medicine.

Nowadays, hiPSCs can be derived from a variety of cellular sources, including umbilical cord blood (14). Significant advancements in reprogramming techniques, such as Sendai virus-mediated reprogramming and non-viral methods, have enhanced the efficiency and safety of hiPSC generation (15). These methods facilitate the conversion of somatic cells into a pluripotent state, thereby expanding the potential for diverse applications in regenerative medicine and personalized therapies. The use of patient-derived hiPSCs can provide large quantities of disease-relevant cells, including even inaccessible ones such as neurons and cardiomyocytes, for disease modeling to study the etiology of human diseases and to develop therapeutic strategies (16). Furthermore, since hiPSCs can be derived from patients themselves, the rapid development of genome editing technologies may allow the correction of disease-causing gene mutations in patient-derived hiPSCs or the introduction of specific mutations into cells from healthy donors (17). Regarding the use of hiPSCs for drug discovery, it is worth mentioning that the identification of a successful therapeutic drug requires a large number of tests and that the failure rate of drug discovery and development is even greater than 90%, resulting in a long, high-risk, and costly process (18). Therefore, the scalability of hiPSC culture and their ability to duplicate indefinitely without undergoing replicative senesce allow disease phenotypes to be consistently recapitulated at a culture scale in order to meet the demands of drug screening (19, 20).

In the field of regenerative medicine, hiPSCs are a promising prospect for cell therapy in a wide range of diseases for which there are currently no cures or effective therapies. The basic paradigm in the use of hiPSCs for cell therapy purposes is to first differentiate them into the desired cell type of interest and then to transplant the resulting specialized tissue-specific cells into patients. The differentiation step is crucial because if undifferentiated hiPSCs are directly injected, they would form tumors (called teratomas) due to their highly proliferative nature and broad differentiation potential (21, 22).

In principle, hiPSCs can be derived directly from individual patients who require therapy; this procedure has the advantage of ensuring an identical immune status and minimizing the risk of transplant rejection. However, producing autologous clinical-grade cells for individual patients would pose major logistical and scientific difficulties, and wide-scale application is likely to be financially prohibitive. An alternative to personalized hiPSC therapy would be to create a bank of clinical-grade hiPSC lines that can be expanded and differentiated for use in a large number of patients (23). Ideally, such a bank would be constituted by hiPSCs obtained from healthy, O-blood-group volunteer donors, selected to maximize the chance of human leukocyte antigens matching and thereby to minimize the risk of allograft rejection. Assuming that a single pluripotent stem cell line can provide cells or tissues for a large number of potential recipients, some studies have been undertaken to calculate the number of hiPSC donors required to cover enough of the population in different countries (1, 23, 24); thus, cells from single donors could potentially last forever and be used by everyone for different purposes.

When a donor signs the informed consent form, it is very important to rigorously explain to him/her all relevant aspects of their own cell donation, including the rights, risks, benefits, research aims, and commercial implications, etc. Moreover, since an individual, from whom hiPSCs have been derived, may want to know about the fate of his/her cells, even the collection of hiPSCs still raises ethical concerns regarding privacy, confidentiality, use, religion, patenting, etc.

In Italy, the ethical debate on stem cells, particularly embryonic ones, is strongly active. Historically, Italy has been a vocal opponent of embryonic stem cell research, considering it morally unacceptable because it involves the destruction of human embryos. Italian law also reflects a conservative stance on embryonic stem cell research: Law 40/2004 restricts the use of human embryos in research and prohibits the cloning and the creation of embryos for research purposes (25). Nevertheless, Italian researchers actively and strongly support stem cell research for basic, translational, and clinical purposes, contributing to global advances in stem cell science.

Despite the fact that stem cell research is permitted and supported in Italy, it raises confusion among the general public, perhaps because it is not easy to understand the major differences between embryonic stem cells and induced pluripotent stem cells. This is an important point because awareness and knowledge regarding hiPSCs can significantly influence the formation of attitudes among the general population and health professionals, which can later be reflected in the course of further research in this field. Thus, surveys to understand the interests of the general public can be a useful vehicle for effectively communicating issues related to hiPSCs. The involvement of citizens and nonspecialist members of the public in gathering opinions on activities related to scientific research occurs mainly through digital technologies, known as “citizen science” (26), and has increased significantly in recent decades (27–32). Although some studies of public opinion on stem cell research have been carried out in the USA, Japan, and Europe (32–35), to our knowledge, no online survey has focused on public attitudes toward hiPSC study participants and recipients as well as possible concerns in the general Italian population.

Based on these premises, the aim of this work was to investigate the knowledge, awareness, and hope in hiPSCs among the Italian adult population using an online survey. In order to obtain a strong and recognized outcome, we decided to use the European online survey management system built for the creation and publication of globally accessible forms to survey the general public. This platform has demonstrated a high consistency and a strong reliability (36).

## 2 Methods

### 2.1 Sample recruitment and study population

The hiPSC web-based survey was implemented using the European Commission’s official open-source management tool EUSurvey, an online platform supported by the European Commission’s ISA program, which promotes interoperability solutions for European public administrations. The survey was available online from January 17, 2021 to June 21, 2021, and it was accessible to everyone. The survey was shared using a snowball sampling strategy (37), mainly through social media platforms (e.g., WhatsApp), mailing lists, and word of mouth, which were chosen as the best communication tools to reach the entire population. The survey was specifically designed to maximize the number of participants not pertaining to the Italian scientific community. To this end, the text of the survey was written, as much as possible, in layman’s language, resulting in a simple structure and quick reading to facilitate understanding and participation. Inclusion criteria were as follows: aged >18 years; had access to a mobile phone, computer, or tablet with internet connection; had the ability to write and speak Italian; and provided online consent to participate in the study.

### 2.2 Variables collected and data transformation

The hiPSC web-based survey was designed as a collaborative project of a working group including biologists, epidemiologists, clinicians, and public health professionals after a comprehensive literature review of existing research (32–34), with the aim of improving knowledge of Italian attitudes toward the use of hiPSCs. Participants were asked to complete the web survey with mandatory questions after reading an introductory page and accepting the option to give consent to participate.

The introductory section defined hiPSCs, focusing on their ability to self-renew and differentiate into several cell types. This section was followed by three comprehension and knowledge questions: (1) This is the first time that I have heard about hiPSCs; (2) hiPSCs obtained from a blood sample can differentiate into all of the mature cell types that constitute the adult human body; (3) hiPSCs obtained from a blood sample can generate undifferentiated daughter cells indefinitely. The possible responses for these three questions were “True,” “False,” and “I do not know.”

The survey then continued with seven questions to assess personal views and possible concerns about hiPSC technology: (4) I would donate a blood sample for the generation of hiPSCs to treat only my relatives, close friends, or myself; (5) I would donate a blood sample for the generation of hiPSCs to treat anyone with an untreatable disease who needed therapy to replace damaged cells or tissues; (6) I would accept that hiPSCs obtained from my blood sample would be used in experiments on animals; (7) I am concerned about the current research and therapeutic applications of these new stem cells; (8) I am concerned about the management of my personal data in relation to the storage and use of the new stem cells derived from my blood cells; (9) I would accept that the new stem cells from my blood might be used for the development of therapies or other applications that are currently unpredictable but regulated; (10) I would donate a blood sample for the generation of hiPSCs, even if they might be acquired in the future by a pharmaceutical company for the development of treatments for incurable diseases. The possible responses to these seven statements were “I agree,” “I disagree,” and “I do not know.”

Finally, the survey included questions to register participants’ personal information, which was then transformed, where appropriate, into the following: gender (men, women, and other); age (18–35, 36–55, and > 55 years); educational level (no university degree vs. university degree); marital status (married, civil union, single, separated/divorced, widow/widower, and I prefer not to answer); employment status (worker with a permanent position, self-employed, worker with a temporary position, unemployed, housewife, retired, and student); work sector (healthcare vs. other); region of origin (northern Italy, central Italy, southern Italy plus islands, and foreign countries); blood donor (current, former, and not a blood donor); willingness to donate organs after death (for organ donation and against organ donation); relatives or friends suffering from rare diseases with no cure (no, experience with relatives and friends, and personal experience); religion (religious vs. nonreligious); frequency of accessing news information (daily, at least once a week, at least once a month, and never); and sources of news information (television, printed newspaper, radio, online newspaper, social networks, and other). The content of the survey is included in Annex 1.

### 2.3 Statistical analysis

The characteristics of the sample were presented using frequencies and percentages. The Chi-squared or Fisher’s exact tests were used to compare the characteristics of participants according to age groups and to possible survey answers. Participants who responded negatively to both comprehension and knowledge (questions 2 and 3) were excluded from further analysis, resulting in a final sample of 1,201 subjects (64.1%). The responses “I do not know,” “disagree,” and “agree” (reference category) were the dependent variables. Tests were not adjusted for multiple comparisons because of the exploratory nature of the study. Analyses were performed using IBM SPSS Statistics for Windows version 25.0 (IBM Corp., Armonk, NY). All *p*-values are two-tailed, and a *p*-value ≤0.05 was considered statistically significant.

### 2.4 Ethics

This project was reviewed by the internal Ethics Committee (Comitato Etico Milano Area 2) and received a favorable opinion (protocol no. 540 on February 26, 2021). The survey study contained only anonymized data from the participants; therefore, the research team had no contact with the participants after their participation in the study. The study was designed, conducted, and reported in accordance with the Declaration of Helsinki, as revised in 2013. Data were handled and stored in accordance with the European Union General Data Protection Regulation (EU GDPR) 2016/679.

## 3 Results

Compared to the Italian population as a whole, the hiPSC study enrolled younger people, especially for those in the following age groups: 18–25 years old (14.3% vs. 9.4%), 26–35 years old (26.2% vs. 12.7%), and > 65 years old (7.5% vs. 26.5%). Meanwhile, the distributions for those aged 36–45, 46–55, and 56–65 years old who completed the study were quite similar to those of the general population. Data on the age distribution of the Italian population were taken from the ISTAT website (38) (Supplementary Figure 1).

Table 1 summarizes the personal characteristics of the 1874 participants who completed the survey by age group. The majority of the sample was aged 36 years or older (59.5%), 63.4% were women (*n* = 1,188), 67.2% had a university degree or postgraduate qualification, and 44.7% were married. Respondents were mostly employed with a stable job (45.2%) and did not work in the health sector (68.3%); 67.9% of participants were from northern Italy. The majority of the sample was not a blood donor (68.3%), and 80.5% were willing to donate their organs after death. Approximately 66% of respondents reported daily access to news information sources, with online newspapers (48.3%) being the main source, followed by television (24.2%). Statistically significant differences were found among age groups.

**Table 1:** Characteristics of the study participants by age group (*n* = 1874).

		Age group (years)								
		18–35 (*n* = 759, 40.5%)		36–55 (*n* = 665, 35.5%)		≥56 (*n* = 450, 24%)		*p*-value	Total (*n* = 1874, 100%)	
		*N*	%	*N*	%	*N*	%		*N*	%
Gender**	Men	271	40.3%	206	30.7%	195	29.0%	<0.001	672	35.9%
	Women	483	40.7%	450	37.9%	255	21.5%		1,188	63.4%
	Other	5	35.7%	9	64.3%	0	0.0%	<0.001	14	0.7%
Education**	No university degree	208	33.8%	214	34.8%	193	31.4%		615	32.8%
	University degree	551	43.8%	451	35.8%	257	20.4%		1,259	67.2%
Marital status**	Married	114	13.6%	400	47.7%	324	38.7%	<0.001	838	44.7%
	Civil union	94	49.5%	81	42.6%	15	7.9%		190	10.1%
	Single	499	80.5%	93	15.0%	28	4.5%		620	33.1%
	Separated/divorced	4	3.3%	68	55.7%	50	41.0%		122	6.5%
	Widow/er	0	0.0%	3	10.3%	26	89.7%		29	1.5%
	I prefer not to answer	48	64.0%	20	26.7%	7	9.3%		75	4.0%
Employment status**	Worker with a permanent position	235	27.7%	429	50.6%	183	21.6%	<0.001	847	45.2%
	Self-employed	62	21.0%	134	45.4%	99	33.6%		295	15.7%
	Worker with a temporary position	160	70.5%	63	27.8%	4	1.8%		227	12.1%
	Unemployed	33	55.9%	19	32.2%	7	11.9%		59	3.1%
	Housewife	2	4.0%	19	38.0%	29	58.0%		50	2.7%
	Retired	0	0.0%	1	0.8%	128	99.2%		129	6.9%
	Student	267	100.0%	0	0.0%	0	0.0%		267	14.2%
Work sector**	Other	469	36.6%	482	37.7%	329	25.7%	<0.001	1,280	68.3%
	Healthcare	290	48.8%	183	30.8%	121	20.4%		594	31.7%
Region**	Northern Italy	495	38.9%	483	37.9%	295	23.2%	<0.001	1,273	67.9%
	Central Italy	105	46.1%	78	34.2%	45	19.7%		228	12.2%
	Southern Italy plus islands	155	44.0%	92	26.1%	105	29.8%		352	18.8%
	Foreign countries	4	19.0%	12	57.1%	5	23.8%		21	1.1%
Are you a blood donor?**	Yes	166	51.6%	110	34.2%	46	14.3%	<0.001	322	17.2%
	In the past	67	24.6%	102	37.5%	103	37.9%		272	14.5%
	No	526	41.1%	453	35.4%	301	23.5%		1,280	68.3%
*Post mortem* organ donation*	Yes	638	42.3%	527	34.9%	344	22.8%	0.003	1,509	80.5%
	No/do not know/I prefer not to answer	121	33.2%	138	37.8%	106	29.0%		365	19.5%
Relatives or friends suffering from rare diseases with no cure*	No	524	38.6%	490	36.1%	344	25.3%	0.034	1,358	72.5%
	Yes. I have had experience with my loved ones	184	46.5%	128	32.3%	84	21.2%		396	21.1%
	Yes. I have had personal experience	51	42.5%	47	39.2%	22	18.3%		120	6.4%
Religion**	Not religious/I prefer not to answer	402	51.5%	239	30.6%	140	17.9%	<0.001	781	41.7%
	Religious	357	32.7%	426	39.0%	310	28.4%		1,093	58.3%
Rate of news information access**	Daily	409	33.2%	479	38.9%	344	27.9%	<0.001	1,232	65.7%
	At least once per week	288	55.0%	152	29.0%	84	16.0%		524	28.0%
	Once per month or less	62	52.5%	34	28.8%	22	18.6%		118	6.3%
Sources of news information**	Online newspaper	380	42.0%	369	40.8%	156	17.2%	<0.001	905	48.3%
	Printed newspaper	8	11.1%	21	29.2%	43	59.7%		72	3.8%
	Radio	26	24.1%	41	38.0%	41	38.0%		108	5.8%
	TV	152	33.6%	140	30.9%	161	35.5%		453	24.2%
	Social network	118	70.2%	37	22.0%	13	7.7%		168	9.0%
	Other	75	44.6%	57	33.9%	36	21.4%		168	9.0%

Row percentages. * means *p*-value <0.05, ** means *p*-value <0.001.

Table 2 shows that compared to those who were unaware of hiPSCs prior to the survey, those who were aware of hiPSCs (question 1) were more likely to be younger, have a university degree, be single, have a temporary job, be a student, work in the health sector, be nonreligious, have a high rate of news information access, and be informed mainly through printed and online newspapers.

**Table 2:** Characteristics of the study participants by response to question 1 “This is the first time that I have heard about hiPSCs” (*n* = 1874).

		False (*n* = 1,013, 54.1%)		Don’t know (*n* = 61, 3.3%)		True (*n* = 800, 42.7%)	
		*N*	%	*N*	%	*N*	%
Gender	Men	366	54.5%	17	2.5%	289	43.0%
	Women	640	53.9%	43	3.6%	505	42.5%
	Other	7	50.0%	1	7.1%	6	42.9%
Age*	18–35 years	449	59.2%	25	3.3%	285	37.5%
	36–55 years	336	50.5%	25	3.8%	304	45.7%
	≥56 years	228	50.7%	11	2.4%	211	46.9%
Education**	No university degree	237	38.5%	28	4.6%	350	56.9%
	University degree	776	61.6%	33	2.6%	450	35.7%
Marital status*	Married	427	51.0%	25	3.0%	386	46.1%
	Civil union	102	53.7%	7	3.7%	81	42.6%
	Single	373	60.2%	22	3.5%	225	36.3%
	Separated/divorced	58	47.5%	5	4.1%	59	48.4%
	Widow/er	15	51.7%	0	0.0%	14	48.3%
	I prefer not to answer	38	50.7%	2	2.7%	35	46.7%
Employment status**	Worker with a permanent position	431	50.9%	31	3.7%	385	45.5%
	Self-employed	144	48.8%	8	2.7%	143	48.5%
	Worker with a temporary position	159	70.0%	5	2.2%	63	27.8%
	Unemployed	23	39.0%	4	6.8%	32	54.2%
	Housewife	18	36.0%	2	4.0%	30	60.0%
	Retired	51	39.5%	4	3.1%	74	57.4%
	Student	187	70.0%	7	2.6%	73	27.3%
Work sector**	Other	518	40.5%	51	4.0%	711	55.5%
	Healthcare	495	83.3%	10	1.7%	89	15.0%
Region	Northern Italy	683	53.7%	41	3.2%	549	43.1%
	Central Italy	122	53.5%	4	1.8%	102	44.7%
	Southern Italy plus islands	197	56.0%	13	3.7%	142	40.3%
	Foreign countries	11	52.4%	3	14.3%	7	33.3%
Blood donor	Yes	192	59.6%	8	2.5%	122	37.9%
	In the past	143	52.6%	9	3.3%	120	44.1%
	No	678	53.0%	44	3.4%	558	43.6%
*Post mortem* organ donation	Yes	835	55.3%	46	3.0%	628	41.6%
	No/do not know/I prefer not to answer	178	48.8%	15	4.1%	172	47.1%
Relatives or friends suffering from rare diseases with no cure	No	718	52.9%	39	2.9%	601	44.3%
	Yes. I have had experience with my loved ones	227	57.3%	18	4.5%	151	38.1%
	Yes. I have had personal experience	68	56.7%	4	3.3%	48	40.0%
Religion*	Not religious/I prefer not to answer	450	57.6%	22	2.8%	309	39.6%
	Religious	563	51.5%	39	3.6%	491	44.9%
Rate of news information access**	Daily	699	56.7%	33	2.7%	500	40.6%
	At least once per week	268	51.1%	19	3.6%	237	45.2%
	Once per month or less	46	39.0%	9	7.6%	63	53.4%
Sources of news information*	Online newspaper	516	57.0%	31	3.4%	358	39.6%
	Printed newspaper	42	58.3%	1	1.4%	29	40.3%
	Radio	56	51.9%	5	4.6%	47	43.5%
	TV	217	47.9%	11	2.4%	225	49.7%
	Social network	78	46.4%	6	3.6%	84	50.0%
	Other	104	61.9%	7	4.2%	57	33.9%

Row percentages.

*p-value≤0.05; **p-value≤0.001.

When we compared the characteristics of the included and excluded participants, we found that those included in the analysis were less likely to not report their gender as well as more likely to have a university degree, to be employed in the healthcare sector, to be nonreligious, and to have a higher rate of news information access (Supplementary Table 1). In addition, the included participants were less likely than the excluded participants to be willing (“I agree”) to donate cells for the treatment of family and friends only (question 4), to be concerned about current research and therapeutic applications of new stem cells (question 7), and to be concerned about the management of their personal data (question 8); meanwhile, they were more likely to agree to their use in animal experiments (question 6) and in the development of therapies or other applications (question 9) (Table 3).

**Table 3:** Comparison of responses between excluded and included participants (*n* = 1874).

		Excluded (*n* = 673, 35.9%)		Included (*n* = 1,201, 64.1%)	
		*N*	%	*N*	%
Question 4: “I would donate a blood sample for the generation of hiPSCs to treat only my relatives, close friends, or myself”**	I disagree	530	78.8%	1,033	86.0%
	Do not know	49	7.3%	63	5.2%
	I agree	94	14.0%	105	8.7%
Question 5: “I would donate a blood sample for the generation of hiPSCs to treat anyone with an untreatable disease who needed therapy to replace damaged cells or tissues”	I disagree	10	1.5%	13	1.1%
	Do not know	38	5.6%	44	3.7%
	I agree	625	92.9%	1,144	95.3%
Question 6: “I would accept that hiPSCs obtained from my blood sample would be used in experiments on animals”*	I disagree	192	28.5%	305	25.4%
	Do not know	160	23.8%	242	20.1%
	I agree	321	47.7%	654	54.5%
Question 7: “I am concerned about the current research and therapeutic applications of these new stem cells”**	I disagree	383	56.9%	857	71.4%
	Do not know	229	34.0%	261	21.7%
	I agree	61	9.1%	83	6.9%
Question 8: “I am concerned about the management of my personal data in relation to the storage and use of the new stem cells derived from my blood cells”**	I disagree	372	55.3%	779	64.9%
	Do not know	147	21.8%	206	17.2%
	I agree	154	22.9%	216	18.0%
Question 9: “I would accept that the new stem cells from my blood might be used for the development of therapies or other applications that are currently unpredictable but regulated”*	I disagree	22	3.3%	18	1.5%
	Do not know	46	6.8%	64	5.3%
	I agree	605	89.9%	1,119	93.2%
Question 10: “I would donate a blood sample for the generation of hiPSCs, even if they might be acquired in the future by a pharmaceutical company for the development of treatments for incurable diseases”	I disagree	174	25.9%	347	28.9%
	Do not know	134	19.9%	201	16.7%
	I agree	365	54.2%	653	54.4%

Column percentages.

*p-value ≤ 0.05; **p-value ≤ 0.001.

**Figure 1:**
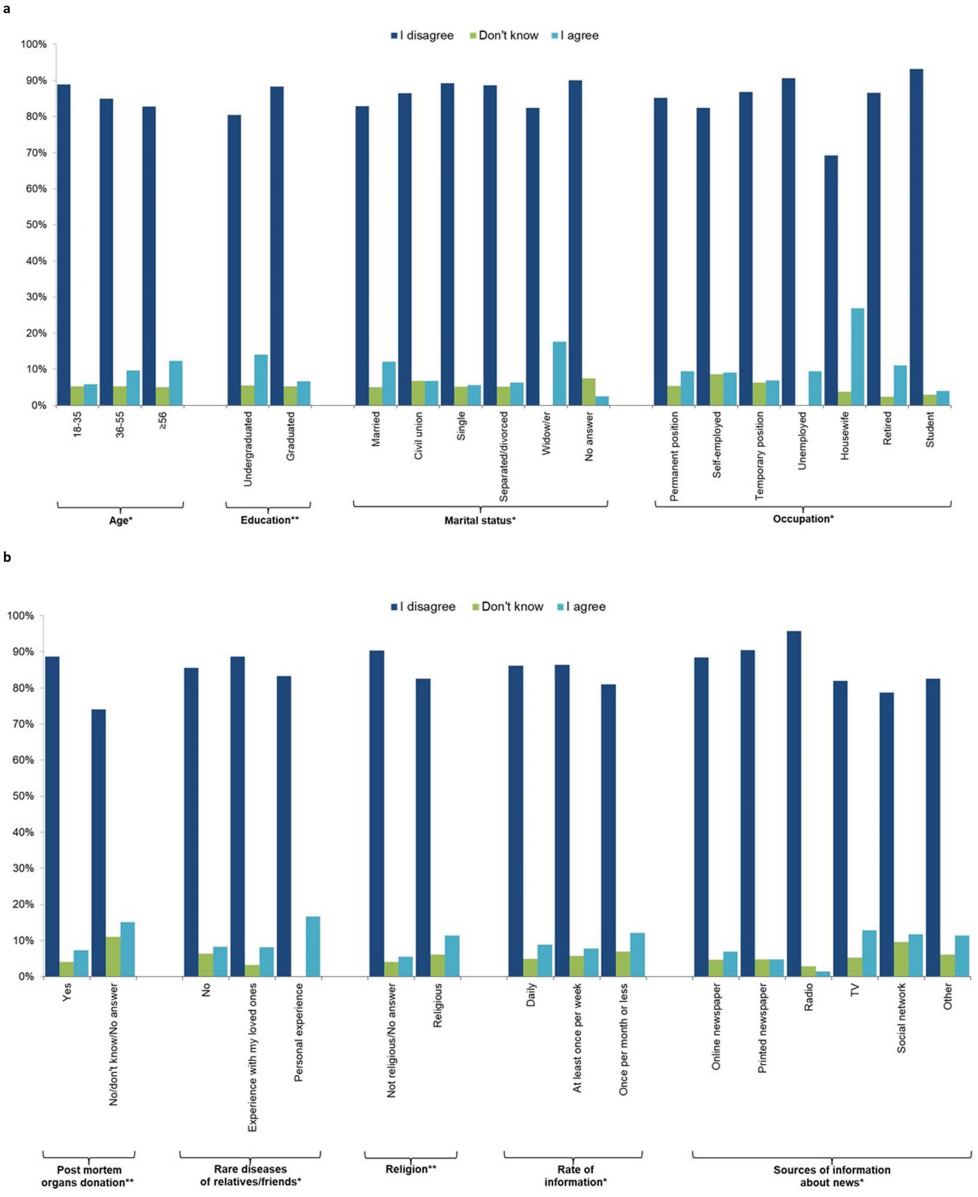
Individual characteristics of the study participants by response to question 4 “I would donate a blood sample for the generation of hiPSCs to treat only my relatives, close friends or me” (*n* = 1,201, 64.1%). **(a)** socio-demographic characteristics, **(b)** behavioural characteristics.

For the subsequent analyses, only the included participants were included as the sample. Figures 1A,B; Supplementary Table 2 shows that the majority of the sample (86.0%) disagreed with statement 4, “I would donate a blood sample for the generation of hiPSCs to treat only my relatives, close friends, or myself.” Younger people (18–35 years) were more likely to disagree than the oldest groups, as were graduates of university compared to those without a university degree, while married people and widowers as well as housewives were more likely to agree. Those in favor of post-mortem organ donation were less likely to support donating their organs only to friends and relatives, as were those with personal experience regarding rare diseases for which there is no treatment. Religious people and those who receive news information once per month or less were more likely to donate only to relatives and friends. Table 4 shows that 95.3% of people agreed that everyone should be treated with these new cells (question 5), with widows being less likely to agree than married/cohabiting, single, and separated/divorced individuals, and post-mortem organ donors being more likely to agree than those who do not wish to donate their organs.

**Table 4:** Characteristics of the study participants by response to question 5 “I would donate a blood sample for the generation of hiPSCs to treat anyone with an untreatable disease who needed therapy to replace damaged cells or tissues” (*n* = 1,201, 64.1%).

		I disagree (*n* = 13, 1.1%)		Don’t know (*n* = 44, 3.7%)		I agree (*n* = 1,144, 95.3%)	
		*N*	%	*N*	%	*N*	%
Gender	Men	7	1.7%	16	3.9%	390	94.4%
	Women	6	0.8%	27	3.4%	750	95.8%
	Other	0	0.0%	1	20.0%	4	80.0%
Age	18–35 years	4	0.8%	20	4.1%	462	95.1%
	36–55 years	3	0.7%	15	3.6%	395	95.6%
	≥56 years	6	2.0%	9	3.0%	287	95.0%
Education	No university degree	6	1.7%	13	3.7%	328	94.5%
	University degree	7	0.8%	31	3.6%	816	95.6%
Marital status*	Married	6	1.1%	19	3.5%	513	95.4%
	Civil union	0	0.0%	6	5.1%	112	94.9%
	Single	3	0.7%	17	4.2%	389	95.1%
	Separated/divorced	1	1.3%	2	2.5%	76	96.2%
	Widow/er	2	11.8%	0	0.0%	15	88.2%
	I prefer not to answer	1	2.5%	0	0.0%	39	97.5%
Employment status	Worker with a permanent position	4	0.7%	21	3.8%	530	95.5%
	Self-employed	3	1.6%	10	5.3%	174	93.0%
	Worker with a temporary position	2	1.4%	4	2.8%	138	95.8%
	Unemployed	0	0.0%	2	6.3%	30	93.8%
	Housewife	1	3.8%	0	0.0%	25	96.2%
	Retired	1	1.2%	3	3.7%	78	95.1%
	Student	2	1.1%	4	2.3%	169	96.6%
Work sector	Other	10	1.3%	33	4.3%	718	94.3%
	Healthcare	3	0.7%	11	2.5%	426	96.8%
Region	Northern Italy	10	1.2%	28	3.4%	781	95.4%
	Central Italy	1	0.7%	6	4.2%	136	95.1%
	Southern Italy plus islands	2	0.9%	9	4.0%	215	95.1%
	Foreign countries	0	0.0%	1	7.7%	12	92.3%
Blood donor	Yes	2	1.0%	5	2.5%	190	96.4%
	In the past	1	0.6%	6	3.5%	164	95.9%
	No	10	1.2%	33	4.0%	790	94.8%
*Post mortem* organ donation**	Yes	7	0.7%	24	2.4%	951	96.8%
	No/do not know/I prefer not to answer	6	2.7%	20	9.1%	193	88.1%
Relatives or friends suffering from rare diseases with no cure	No	7	0.8%	31	3.6%	831	95.6%
	Yes. I have had experience with my loved ones	5	2.0%	12	4.8%	231	93.1%
	Yes. I have had personal experience	1	1.2%	1	1.2%	82	97.6%
Religion	Not religious/I prefer not to answer	5	0.9%	16	3.0%	511	96.1%
	Religious	8	1.2%	28	4.2%	633	94.6%
Rate of news information access	Daily	10	1.2%	28	3.5%	773	95.3%
	At least once per week	1	0.3%	13	3.9%	318	95.8%
	Once per month or less	2	3.4%	3	5.2%	53	91.4%
Sources of news information	Online newspaper	4	0.7%	19	3.2%	570	96.1%
	Printed newspaper	1	2.4%	1	2.4%	40	95.2%
	Radio	0	0.0%	1	1.4%	69	98.6%
	TV	4	1.4%	13	4.5%	271	94.1%
	Social network	1	1.1%	5	5.3%	88	93.6%
	Other	3	2.6%	5	4.4%	106	93.0%

Row percentages.

*p-value ≤ 0.05; **p-value ≤ 0.001.

**Figure 2:**
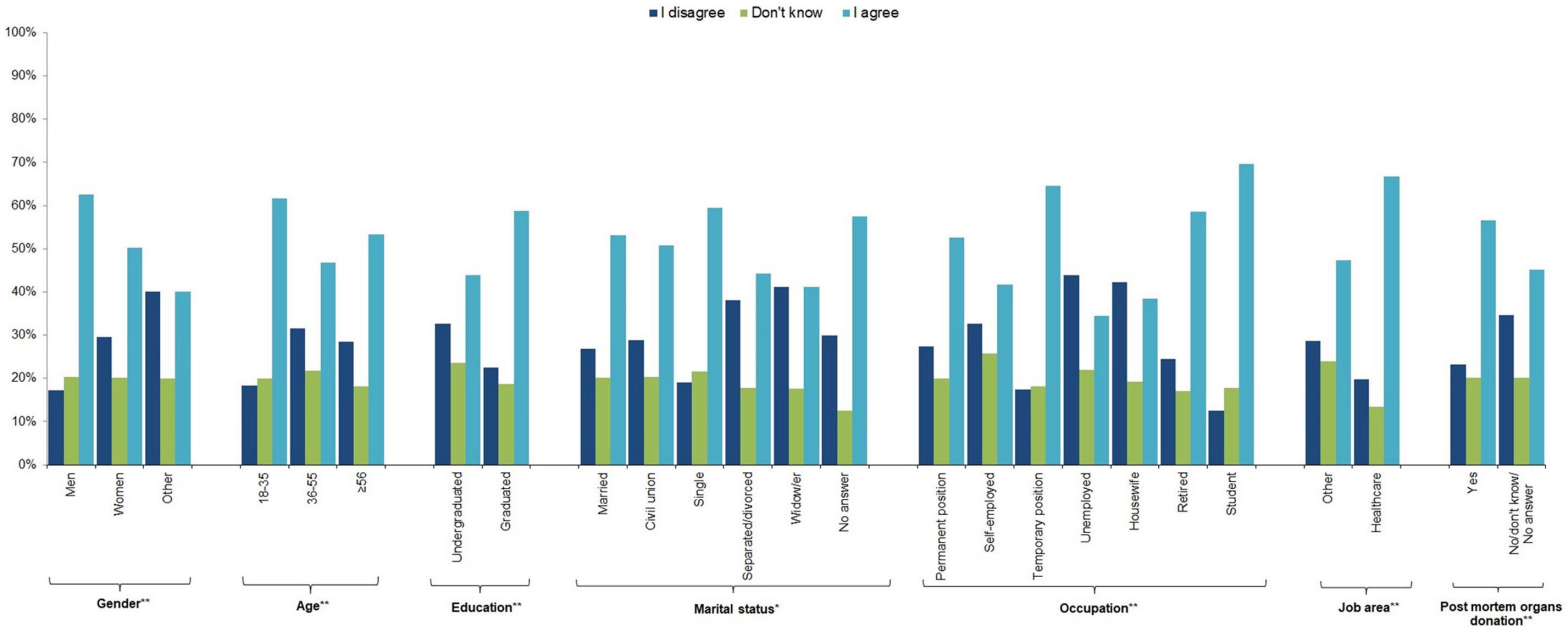
Individual characteristics of the study participants by response to question 6 “I would accept that hiPSCs obtained from my blood sample would be used in experiments on animals” (*n* = 1,201, 64.1%).

In response to question 6 on the use of these cells in animal experiments, just over half (54.5%) of the sample agreed, with the other half split between those who disagreed (25.4%) and those who answered “I do not know” (20.1%). Men, younger people, university graduates, people with a temporary job, students, people working in the health sector, and post-mortem organ donors were more likely to agree, as were single people and those who preferred not to answer (*p*-value≤0.05) (Figure 2; Supplementary Table 3).

**Figure 3:**
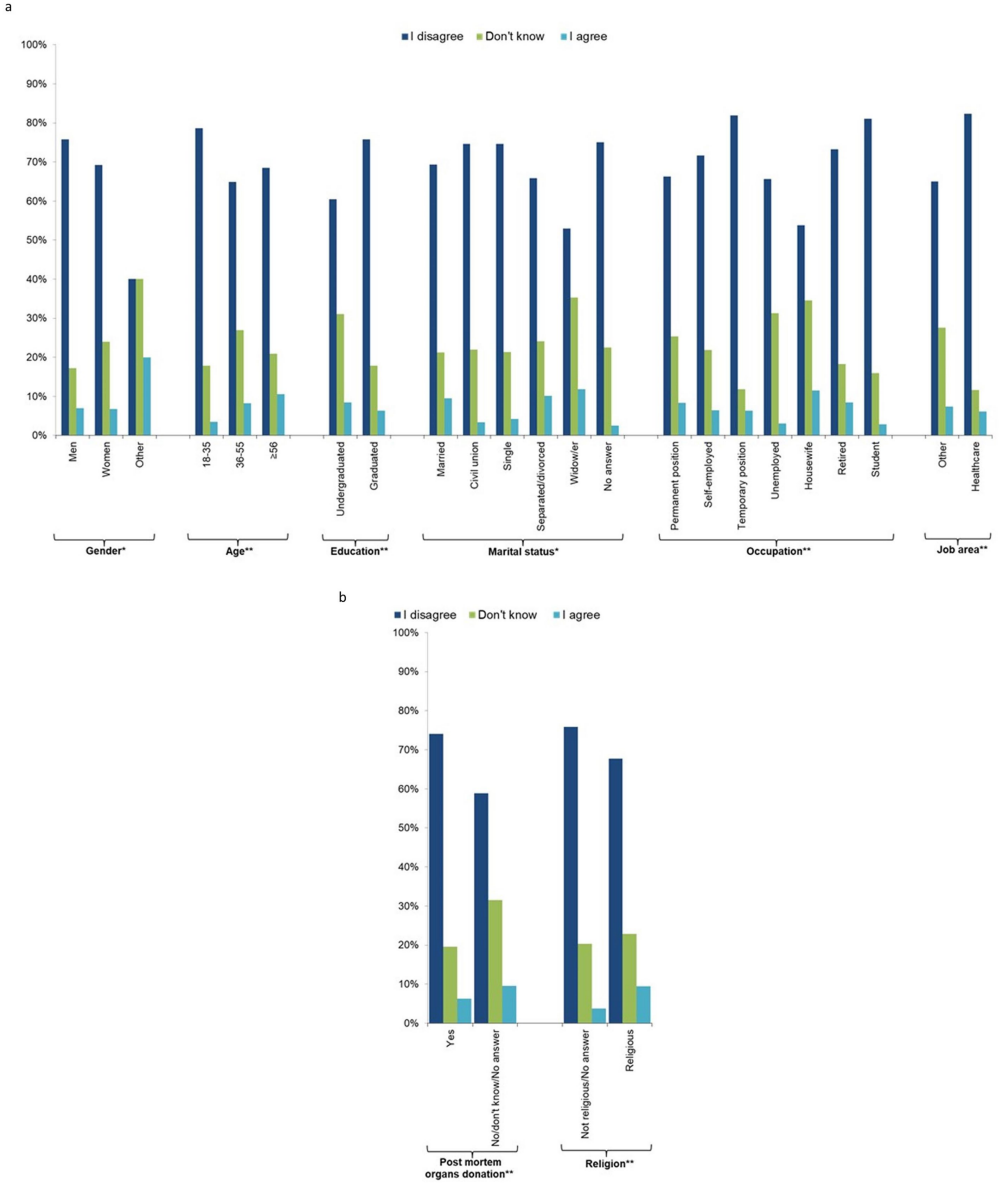
Individual characteristics of the study participants by response to question 7 “I am concerned about the current research and therapy applications of these new stem cells” (*n* = 1,201, 64.1%). **(a)** socio-demographic characteristics, **(b)** behavioural characteristics.

Those most concerned about current research and therapeutic applications of new stem cells (question 7, Figures 3A,B; Supplementary Table 4) were the minority of the sample (6.9%), and 21.7% answered “I do not know.” The most concerned people were those who did not indicate their gender, older people (≥56 years), separated/divorced or widowed people, housewives, people not working in the health sector, those not willing to donate their organs after death, and religious people.

**Figure 4:**
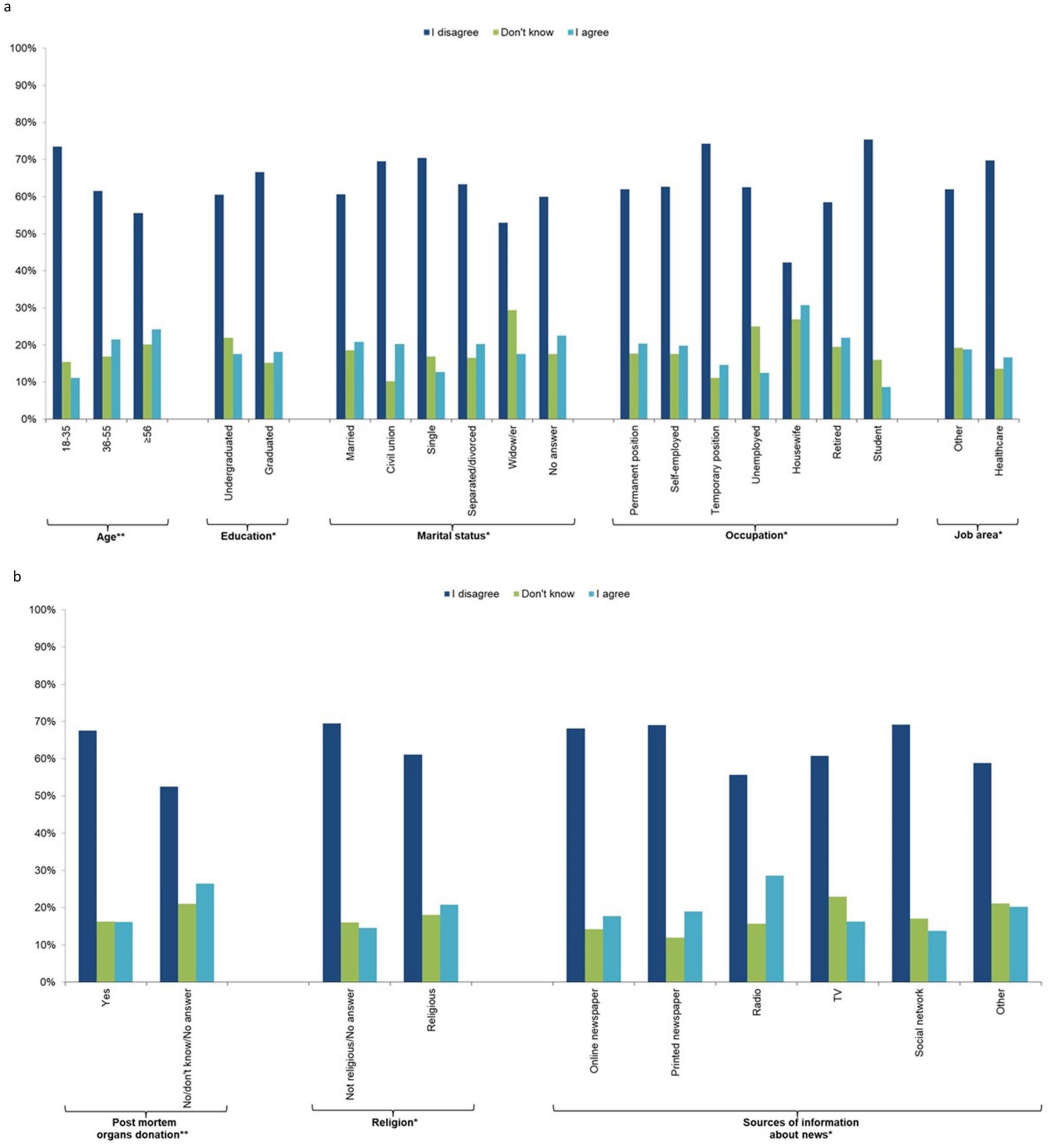
Individual characteristics of the study participants by response to question 8 “I am concerned about the management of my personal data in relation to storage and use of the new stem cells derived from my blood cells” (*n* = 1,201, 64.1%). **(a)** socio-demographic characteristics, **(b)** behavioural characteristics.

The majority (65%) of the sample was not concerned about the management of their personal data (question 8), 17.2% did not know, and 18.0% were concerned. There was a decreasing trend with age, with the youngest (18–35 years) being less concerned than those aged ≥56 years. In addition, university graduates, those who preferred not to provide their marital status, widow/ers, housewives, retired people, those not working in the health sector, religious people, those who would not donate or were uncertain about post-mortem organ donation, and those who get their news from the radio were more concerned about the management of their personal data (Figure 4; Supplementary Table 5).

Participants who responded positively to question 9, “I would accept that the new stem cells from my blood might be used for the development of therapies or other applications that are currently unpredictable but regulated” (Table 5), were almost the whole sample (93.2%). People who lived in the regions of southern Italy, on the islands, or abroad and who were not post-mortem organ donors were more likely to disagree or respond “I do not know.”

**Table 5:** Characteristics of the study participants by response to question 9 “I would accept that the new stem cells from my blood might be used for the development of therapies or other applications that are currently unpredictable but regulated” (*n* = 1,201. 64.1%).

		I disagree (*n* = 18, 1.5%)		Do not know (*n* = 64, 5.3%)		I agree (*n* = 1,119, 93.2%)	
		*N*	%	*N*	%	*N*	%
Gender	Men	7	1.7%	21	5.1%	385	93.2%
	Women	11	1.4%	43	5.5%	729	93.1%
	Other	0	0.0%	0	0.0%	5	100.0%
Age	18–35 years	4	0.8%	22	4.5%	460	94.7%
	36–55 years	8	1.9%	30	7.3%	375	90.8%
	≥56 years	6	2.0%	12	4.0%	284	94.0%
Education	No university degree	5	1.4%	18	5.2%	324	93.4%
	University degree	13	1.5%	46	5.4%	795	93.1%
Marital status	Married	11	2.0%	31	5.8%	496	92.2%
	Civil union	3	2.5%	4	3.4%	111	94.1%
	Single	2	0.5%	22	5.4%	385	94.1%
	Separated/divorced	1	1.3%	3	3.8%	75	94.9%
	Widow/er	0	0.0%	2	11.8%	15	88.2%
	I prefer not to answer	1	2.5%	2	5.0%	37	92.5%
Employment status	Worker with a permanent position	9	1.6%	30	5.4%	516	93.0%
	Self-employed	3	1.6%	12	6.4%	172	92.0%
	Worker with a temporary position	2	1.4%	8	5.6%	134	93.1%
	Unemployed	0	0.0%	2	6.3%	30	93.8%
	Housewife	1	3.8%	1	3.8%	24	92.3%
	Retired	2	2.4%	3	3.7%	77	93.9%
	Student	1	0.6%	8	4.6%	166	94.9%
Work sector	Other	13	1.7%	47	6.2%	701	92.1%
	Healthcare	5	1.1%	17	3.9%	418	95.0%
Region*	Northern Italy	16	2.0%	46	5.6%	757	92.4%
	Central Italy	1	0.7%	8	5.6%	134	93.7%
	Southern Italy plus islands	1	0.4%	7	3.1%	218	96.5%
	Foreign countries	0	0.0%	3	23.1%	10	76.9%
Blood donor	Yes	4	2.0%	5	2.5%	188	95.4%
	In the past	2	1.2%	10	5.8%	159	93.0%
	No	12	1.4%	49	5.9%	772	92.7%
*Post mortem* organ donation**	Yes	12	1.2%	40	4.1%	930	94.7%
	No/do not know/I prefer not to answer	6	2.7%	24	11.0%	189	86.3%
Relatives or friends suffering from rare diseases with no cure	No	11	1.3%	45	5.2%	813	93.6%
	Yes. I have had experience with my loved ones	5	2.0%	14	5.6%	229	92.3%
	Yes. I have had personal experience	2	2.4%	5	6.0%	77	91.7%
Religion	Not religious/I prefer not to answer	4	0.8%	23	4.3%	505	94.9%
	Religious	14	2.1%	41	6.1%	614	91.8%
Rate of news information access	Daily	12	1.5%	37	4.6%	762	94.0%
	At least once per week	5	1.5%	24	7.2%	303	91.3%
	Once per month or less	1	1.7%	3	5.2%	54	93.1%
Sources of news information	Online newspaper	7	1.2%	29	4.9%	557	93.9%
	Printed newspaper	1	2.4%	4	9.5%	37	88.1%
	Radio	1	1.4%	4	5.7%	65	92.9%
	TV	2	0.7%	19	6.6%	267	92.7%
	Social network	2	2.1%	3	3.2%	89	94.7%
	Other	5	4.4%	5	4.4%	104	91.2%

Row percentages.

*p-value ≤ 0.05; **p-value ≤ 0.001.

Table 6 shows that just over half of the sample (54.4%) agreed to donate a blood sample for the generation of hiPSCs, even if they might be purchased in the future by a pharmaceutical company for the development of treatments for incurable diseases (question 10). People who did not specify their gender, those aged ≥56 years, those who were not willing to donate their organs after death, and those who got their information from printed newspapers or other sources were more likely to disagree or respond “I do not know.”

**Table 6:** Characteristics of the study participants by response to question 10 “I would donate a blood sample for the generation of hiPSCs, even if they might be acquired in the future by a pharmaceutical company for the development of treatments for incurable diseases” (*n* = 1,201. 64.1%).

		I disagree (*n* = 347, 28.9%)		Do not know (*n* = 201, 16.7%)		I agree (*n* = 653, 54.4%)	
		*N*	%	*N*	%	*N*	%
Gender**	Men	138	33.4%	49	11.9%	226	54.7%
	Women	208	26.6%	149	19.0%	426	54.4%
	Other	1	20.0%	3	60.0%	1	20.0%
Age**	18–35 years	110	22.6%	82	16.9%	294	60.5%
	36–55 years	133	32.2%	78	18.9%	202	48.9%
	≥56 years	104	34.4%	41	13.6%	157	52.0%
Education	No university degree	104	30.0%	59	17.0%	184	53.0%
	University degree	243	28.5%	142	16.6%	469	54.9%
Marital status	Married	167	31.0%	83	15.4%	288	53.5%
	Civil union	26	22.0%	21	17.8%	71	60.2%
	Single	100	24.4%	75	18.3%	234	57.2%
	Separated/divorced	31	39.2%	11	13.9%	37	46.8%
	Widow/er	7	41.2%	3	17.6%	7	41.2%
	I prefer not to answer	16	40.0%	8	20.0%	16	40.0%
Employment status	Worker with a permanent position	162	29.2%	91	16.4%	302	54.4%
	Self-employed	58	31.0%	35	18.7%	94	50.3%
	Worker with a temporary position	44	30.6%	28	19.4%	72	50.0%
	Unemployed	11	34.4%	5	15.6%	16	50.0%
	Housewife	5	19.2%	5	19.2%	16	61.5%
	Retired	33	40.2%	8	9.8%	41	50.0%
	Student	34	19.4%	29	16.6%	112	64.0%
Work sector	Other	216	28.4%	128	16.8%	417	54.8%
	Healthcare	131	29.8%	73	16.6%	236	53.6%
Region	Northern Italy	225	27.5%	144	17.6%	450	54.9%
	Central Italy	48	33.6%	21	14.7%	74	51.7%
	Southern Italy plus islands	68	30.1%	34	15.0%	124	54.9%
	Foreign countries	6	46.2%	2	15.4%	5	38.5%
Blood donor	Yes	57	28.9%	30	15.2%	110	55.8%
	In the past	44	25.7%	28	16.4%	99	57.9%
	No	246	29.5%	143	17.2%	444	53.3%
*Post mortem* organ donation**	Yes	271	27.6%	153	15.6%	558	56.8%
	No/do not know/I prefer not to answer	76	34.7%	48	21.9%	95	43.4%
Relatives or friends suffering from rare diseases with no cure	No	256	29.5%	146	16.8%	467	53.7%
	Yes. I have had experience with my loved ones	72	29.0%	43	17.3%	133	53.6%
	Yes. I have had personal experience	19	22.6%	12	14.3%	53	63.1%
Religion	Not religious/I prefer not to answer	148	27.8%	79	14.8%	305	57.3%
	Religious	199	29.7%	122	18.2%	348	52.0%
Rate of news information access	Daily	234	28.9%	129	15.9%	448	55.2%
	At least once per week	97	29.2%	58	17.5%	177	53.3%
	Once per month or less	16	27.6%	14	24.1%	28	48.3%
Sources of news information*	Online newspaper	158	26.6%	111	18.7%	324	54.6%
	Printed newspaper	16	38.1%	3	7.1%	23	54.8%
	Radio	17	24.3%	17	24.3%	36	51.4%
	TV	82	28.5%	50	17.4%	156	54.2%
	Social network	27	28.7%	11	11.7%	56	59.6%
	Other	47	41.2%	9	7.9%	58	50.9%

Row percentages.

*p-value≤0.05; **p-value≤0.001.

## 4 Discussion

### 4.1 Summary of main findings

hiPSCs have great promise for cell therapy in the field of regenerative medicine, offering a cure or effective treatment for a variety of diseases for which there are currently no viable treatments. Combining the best aspects of hESCs and adult stem cells, hiPSCs circumvent most ethical controversies because they are generated without the use of embryos or oocytes. Importantly, hiPSCs offer an almost limitless supply of stem cells because of their ability to differentiate into any type of cell and their potential to be propagated indefinitely (17).

The present study provides a snapshot of the characteristics of an Italian sample aimed at exploring knowledge, awareness, and hope in hiPSCs. To the best of our knowledge, this is the first online survey to investigate public attitudes and concerns regarding hiPSC study participants and recipients in the general Italian population.

Looking at the responses to the survey, in the first question on knowledge of hiPSCs, 54.1% of Italian respondents had heard of hiPSCs. This is interesting, but it is worth noting that 67.2% of our respondents were university graduates and 31.7% worked in the health sector; perhaps this can explain the high percentage of people who were aware of hiPSCs.

A similar question was posed by Shineha et al. to respondents from six different countries. The authors surveyed knowledge of hiPSCs in France, Germany, the UK, the USA, South Korea, and Japan among 600 respondents, 100 from each country. In Germany and the UK, only 12 and 15% of respondents knew about hiPSCs, respectively. In France, the USA, and South Korea, 30% of respondents were aware of hiPSCs, while 97% of Japanese respondents were aware of hiPSCs. This result is remarkable, showing that the population of Japan has been educated about this type of cell and its use in research and therapy since its discovery in 2007 by Professor Yamanaka in their country (29).

As expected, those who had heard about hiPSCs were more likely to be young, to have a university degree, to have a temporary job, to be a student, to work in the health sector, and to have a high rate of news information access. The demographic information regarding education and work sectors provided predictable results, as people with a university degree and those working in the health sector were likely to have studied these cell types during their lives. It should be noted that people with a high rate of news information access also were more aware of these cells, even though they may never have heard of it at university or school.

A survey conducted by Ishihara et al. examined the awareness and interest in hiPSCs and regenerative medicine among 2,396 Japanese high school and university students. The results showed that over 80% of the students recognized hiPSCs and regenerative medicine. However, many of them expressed concerns about the side effects, safety, and costs associated with regenerative treatments as well as supported the need for enhanced education to improve understanding of hiPSCs (39). These results are consistent with our findings, although, again, the level of knowledge in Japan may be influenced by Professor Yamanaka’s Nobel Prize achievement.

The majority of the sample (86.0%) disagreed with the statement “I would donate a blood sample for the generation of hiPSCs to treat only my relatives, close friends, or myself.” However, 95.3% of people agreed that everyone, not just relatives or close friends, should be treated with these new cells, and they would donate them to anyone. This is the key finding of our work: people, regardless of their level of knowledge about stem cells, would donate cells to treat anyone who needed them due to incurable diseases.

When asked about the use of hiPSCs in animal experiments, just over half of respondents agreed, while the rest were evenly divided between those who disagreed and those who were unsure. It should be noted that the latter group still has a chance to understand the importance of animal testing in the development of new therapies via education. For example, even if innovative three-dimensional models are becoming increasingly used in drug screening (40, 41), animal testing remains a crucial part of the research process, providing valuable insights that cannot yet be fully replicated by alternative methods. In fact, regulatory requirements often mandate animal testing to ensure the safety and efficacy of new drugs and treatments before human trials.

Nevertheless, question 6 reflects the issues surrounding the use of animals in scientific research, with ethical, scientific, and social concerns, as well as active involvement of the general public and animal rights activist associations. Recently, Bearth and colleagues conducted a survey aimed at investigating public views on animal testing and potential alternatives in Switzerland, and they showed that animal testing was accepted mainly for medicines and food (42).

There have always been conflicting opinions on the issue of animal testing, with major problems raised by animal rights associations; in addition, public debate has often suffered from misleading information that is disseminated by individuals or groups who oppose animal testing (43).

In Italy, for example, a neuroscientist at the University of Turin and Tilburg University faced controversy with animal rights activists over his research using primates to study visual perception and cortical blindness. Despite these challenges, he defended the scientific need for animal models to understand neurological diseases and to develop therapies. This case highlights the wider debate between the scientific community and animal rights movements over animal experimentation (44).

Moving forward to question 7, 65% of the sample population was not concerned about the management of their personal data. In general, there was a decreasing trend with age, with the youngest being less concerned than those aged ≥56 years. This is an interesting point, given the current importance of managing personal data in the age of social media.

In the last question, we found that just over half of the sample population would agree to donate a blood sample for the generation of hiPSCs, even if they might be purchased in the future by a pharmaceutical company for the development of treatments for incurable diseases. It is important to note that while 1,144 respondents agreed with the statement “I would donate a blood sample for the generation of hiPSCs to treat anyone with an untreatable disease who needed therapy to replace damaged cells or tissues,” when the role and profit of pharmaceutical companies come into play, this number drops to 653. This finding indicates that 42.9% of those who said they would be willing to donate to anyone would reconsider their position if a pharmaceutical corporation got involved.

Thus, we identified specific subgroups of people who were more concerned and skeptical about hiPSCs, such as older, less educated, non-blood donors and religious individuals. In fact, we found that post-mortem organ donors were more likely to agree that stem cells should be donated to everyone, used to treat incurable diseases, and used in animal experiments than nondonors, who were more concerned about the use and handling of their personal data and future applications. Therefore, we hypothesized that those who choose to donate their organs are by nature altruistic toward other people and very knowledgeable about the progress of science. Similarly, we observed that young people often agreed more with the use of these cells and, in general, had fewer concerns regarding this technology than the older age groups. Again, we speculated that young people may be more receptive to the use of new technologies, even in the field of healthcare.

### 4.2 Limitations and strengths of the study

This study had some limitations that must be addressed. For example, there was intrinsic selection bias in this study due to the voluntary participation in the hiPSC web-based survey. Although the high level of participation allowed us to obtain a fairly balanced sample, some of the characteristics of the sample were not sufficiently representative of the Italian adult population as would be expected in a web survey using snowball sampling. In particular, as this method does not use random selection, it is prone to error and sample bias because not all members of a group have an equal chance of being selected and participants are likely to involve people who are similar to themselves (37). Indeed, we found an over-representation of women, young respondents with high levels of education, and participants from the healthcare sector. Furthermore, the majority of respondents (67.9%) were from northern Italy, which may not represent the views of the whole country, especially the south, as there are significant regional differences for historical, socio-cultural and administrative reasons that may have influenced public attitudes. In particular, based on previous research (45–47), we can speculate that this geographical bias may have led to an overestimation of the percentage of general attitudes toward willingness to donate blood samples for hiPSC generation in the present sample compared to the general population.

Therefore, as the sample was self-selected, the results should be generalized with caution.

Future research should take these limitations into account by opting for different recruitment approaches (e.g., using simple random sampling to select a random set of participants from a large population in which everyone has an equal chance of being selected), or by following a set of best practices to increase the reliability of snowball sampling research.

Moreover, the cross-sectional and observational design of the study provides only a snapshot of the community at the time of the survey and it is not possible to draw causal inferences.

Another weakness of this study is that the data were self-reported, which may have introduced measurement error and recall bias (e.g., misunderstanding of the survey questions, etc.). However, it is reasonable to assume that non-differential misclassification may have occurred, where the likelihood of misclassifying exposure is independent of the possible survey responses (outcomes) and vice versa, increasing the similarity between the exposed and unexposed groups. In addition, the use of an anonymous online survey (no personal contact and no identifying information), significantly reduced the likelihood of social desirability bias, which is the tendency of respondents to answer in a way that is more socially acceptable to others.

Nevertheless, the hiPSC web survey has several strengths. First, to the best of our knowledge, this is the first online survey to focus on public attitudes toward hiPSCs and possible concerns in the general Italian population. Second, it reached a large sample of adults covering all regions of Italy, which provides a robust dataset for statistical analysis. Third, the survey was designed to take into account some information on sociodemographic characteristics and behaviors based on previous literature reports adding depth to the data interpretation. Fourth, the survey was implemented using EUSurvey, an open-source management tool developed by the European Commission that guarantees complete anonymity of participants and their personal data as well as a high level of data security.

Fifthly, the study draws comparisons with similar research carried out in other countries, such as Japan, the USA and Sweden. This adds an international perspective and increases the relevance of the finding.

Finally, the use of web-based surveys as an alternative method for obtaining public opinion on various public health issues can overcome the higher costs and interviewer bias typical of traditional methods (e.g., telephone surveys), as they are a cheap and scalable means of efficiently and rapidly involving a large number of people in a study, regardless of geographical distance (48).

## 5 Conclusion

In conclusion, this work demonstrates that people are generally willing to donate cells for the development of new therapies to cure incurable diseases, but some are concerned about data management, animal testing, and potential profits for pharmaceutical companies; these concerns are particularly evident in some specific subgroups of individuals. Furthermore, this work highlights the importance of surveys based on citizen engagement to accurately identify groups of individuals who are the least receptive to certain topics. Such insights can enable the precise targeting and dissemination of campaigns designed to elevate awareness of sensitive research issues.

## Data Availability

The original contributions presented in the study are included in the article/Supplementary material, further inquiries can be directed to the corresponding author.
