# Supplementary material for: Public attitudes toward the use of human induced pluripotent stem cells: insights from an Italian adult population
Source: Front Public Health. 2024 Nov 6;12:1491257. doi: 10.3389/fpubh.2024.1491257 (PMC11576450; doi:10.3389/fpubh.2024.1491257)
Supplement: Supplementary file 1 [file Data_Sheet_1.docx]

*Are stem cells in your future?*

The purpose of this short questionnaire is to have your opinion about the possibility of obtaining stem cells from a blood sample that you can donate for the treatment of diseases that currently have no cure. Your answers will contribute to the establishment of a more direct dialogue between researchers in this field and the Italian population.

*What are human induced pluripotent stem cells?*

Our body is made of many different cell types, each with specialized functions that contribute to the formation and functionality of tissues and organs. These unique cell functions allow the heart to beat, the brain to think, the kidneys to purify blood, the skin to regenerate, etc. These are defined as specialized cells; once they became specialized, they are no longer capable of being stem cells, so they are not able to generate new specialized cells and new tissues anymore.

In the adult human body, some types of stem cells are still present, but they are limited in their ability to generate daughter cells. Today, thanks to the help of advanced cell biology techniques (reprogramming), it is possible to obtain so-called “induced pluripotent stem cells.” These new stem cells regain the capacity to generate daughter cells indefinitely and are able to specialize into any type of cell that is present in the organism.

New tissues and organs can be literally created starting from these stem cells, thus offering hope for the treatment of diseases that require regeneration or replacement of tissues.

*What could happen to your cells?*

After the donation of a blood sample, your cells will be reprogrammed to obtain induced pluripotent stem cells capable of generating daughter cells for a potentially unlimited time. The new stem cells will be stored in a certified public biobank and will be used in complete anonymity, without any financial return to you. These stem cells will be used both for research purposes and for use in clinical studies in humans.

*Questions*

*I declare

- To be more than 18 years old
- To have read the information above about human induced pluripotent stem cells (hiPSCs)
- To agree to participate in this survey

1. This is the first time that I have heard about hiPSCs.

- True
- False
- I don’t know

2. hiPSCs obtained from a blood sample can differentiate into all of the mature cell types that constitute the adult human body.

- True
- False
- I don’t know

3. hiPSCs obtained from a blood sample can generate undifferentiated daughter cells indefinitely.

- True
- False
- I don’t know

4. I would donate a blood sample for the generation of hiPSCs to treat only my relatives, close friends, or myself.

- I agree
- I disagree
- I don’t know

5. I would donate a blood sample for the generation of hiPSCs to treat anyone with an untreatable disease who needed therapy to replace damaged cells or tissues.

- I agree
- I disagree
- I don’t know

6. I would accept that hiPSCs obtained from my blood sample would be used in experiments on animals.

- I agree
- I disagree
- I don’t know

7. I am concerned about the current research and therapeutic applications of these new stem cells.

- I agree
- I disagree
- I don’t know

8. I am concerned about the management of my personal data in relation to the storage and use of the new stem cells derived from my blood cells.

- I agree
- I disagree
- I don’t know

9. I would accept that the new stem cells might be used for the development of therapies or other applications that are currently unpredictable but regulated.

- I agree
- I disagree
- I don’t know

10. I would donate a blood sample for the generation of hiPSCs, even if they might be acquired in the future by a pharmaceutical company for the development of treatments for incurable diseases.

- I agree
- I disagree
- I don’t know

Personal information

1. How old are you?

- 18–25 years old
- 26–35 years old
- 36–45 years old
- 46–55 years old
- 56–65 years old
- >65 years old

1. How would you describe your gender?

- Man
- Woman
- Other
- I prefer not to answer

1. What region are you from?

- Abruzzo
- Basilicata
- Campania
- Calabria
- Emilia-Romagna
- Friuli-Venezia-Giulia
- Liguria
- Lazio
- Lombardia
- Marche
- Molise
- Piemonte
- Puglia
- Sardegna
- Sicilia
- Toscana
- Trentino-Alto Adige
- Umbria
- Valle d’Aosta
- Veneto
- Abroad

1. How would you describe your marital status?

- Married
- Single
- I prefer not to answer
- Separated/divorced
- Civil union
- Widow/er

5. What is your highest level of education?

- No qualification
- Primary school
- Secondary school
- High school
- Bachelor’s degree
- Master’s degree
- Postgraduate level of education

1. What is your employment status?

- Retired
- Housewife
- Student
- Unemployed
- Worker with a temporary position
- Worker with a permanent position
- Self-employed

1. What is your area of employment?

- Environment/agriculture/livestock farming
- Private/public/state administration
- Building/town planning
- Training/education
- Finance/legal/consulting
- Industry/craft/enterprise
- Media/digital/information technology
- Health/pharmaceutical
- Culture/art/entertainment/sport
- Tourism/transport
- Food/restaurant
- Sales/marketing
- Non-profit/social services
- No profession

1. Are you a blood donor?

- Yes
- No
- In the past

1. Would you donate your organs post mortem?

- Yes
- No
- I don’t know
- I prefer not to answer

1. Do you or someone close to you (relatives or friends) suffer from rare diseases or pathological conditions that do not respond to conventional treatments or for which there is no cure?

- No
- Yes, I have had personal experience
- Yes, I have had experience with my loved ones

1. How would you describe your religious orientation?

- Catholic Christian
- Orthodox Christian
- Protestant
- Christian Jehovah’s Witness
- Other Christian religions
- Muslim
- Hindu
- Buddhist
- Jew
- Sikh
- Other Eastern religions
- Other religions
- Agnostic/atheist
- I prefer not to answer

1. What is your rate of news information access?

- Daily
- At least once per week
- Once per month or less

1. What are your sources of news information?

- Online newspaper
- Printed newspaper
- Radio
- TV
- Social network
- Other
