# Supplementary material for: Public attitudes toward the use of human induced pluripotent stem cells: insights from an Italian adult population
Source: Front Public Health. 2024 Nov 6;12:1491257. doi: 10.3389/fpubh.2024.1491257 (PMC11576450; doi:10.3389/fpubh.2024.1491257)
Supplement: Supplementary file 2 [file Data_Sheet_2.docx]

**Supplementary Figure 1. Age distribution of hiPSCs web survey participants compared to the Italian population at the January 1, 2021.**

**
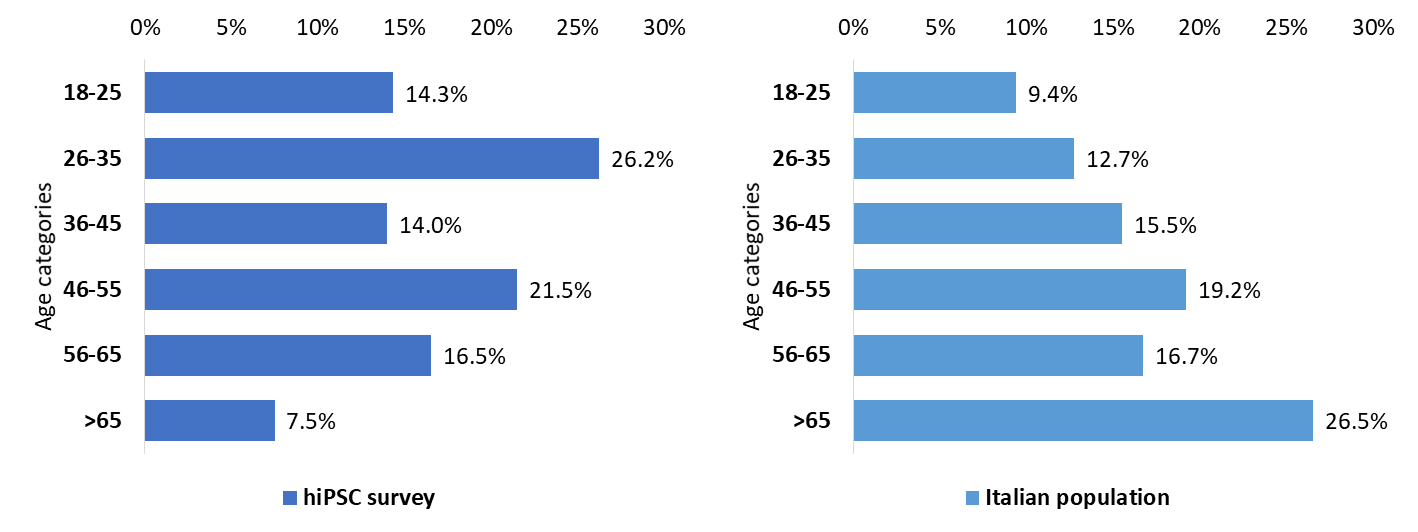
**

**Supplementary Table 1. Comparison of study characteristics between excluded and included participants (n=1874)**

|  | | **Excluded**  **(n=673, 35.9%)** | | **Included**  **(n=1201, 64.1%)** | |
| --- | --- | --- | --- | --- | --- |
|  |  | **N** | **%** | **N** | **%** |
| **Gender*** | Men | 259 | 38.5% | 413 | 61.5% |
|  | Women | 405 | 34.1% | 783 | 65.9% |
|  | Other | 9 | 64.3% | 5 | 35.7% |
| **Age** | 18-35 | 273 | 36.0% | 486 | 64.0% |
|  | 36-55 | 252 | 37.9% | 413 | 62.1% |
|  | ≥56 | 148 | 32.9% | 302 | 67.1% |
| **Education**** | Undergraduated | 268 | 43.6% | 347 | 56.4% |
|  | Graduated | 405 | 32.2% | 854 | 67.8% |
| **Marital status** | Married | 300 | 35.8% | 538 | 64.2% |
|  | Civil union | 72 | 37.9% | 118 | 62.1% |
|  | Single | 211 | 34.0% | 409 | 66.0% |
|  | Separated/divorced | 43 | 35.2% | 79 | 64.8% |
|  | Widow/er | 12 | 41.4% | 17 | 58.6% |
|  | I prefer do not answer | 35 | 46.7% | 40 | 53.3% |
| **Occupation** | Worker with permanent position | 292 | 34.5% | 555 | 65.5% |
|  | Self-employed | 108 | 36.6% | 187 | 63.4% |
|  | Worker with temporary position | 83 | 36.6% | 144 | 63.4% |
|  | Unemployed | 27 | 45.8% | 32 | 54.2% |
|  | Housewife | 24 | 48.0% | 26 | 52.0% |
|  | Retired | 47 | 36.4% | 82 | 63.6% |
|  | Student | 92 | 34.5% | 175 | 65.5% |
| **Job area**** | Other | 519 | 40.5% | 761 | 59.5% |
|  | Healthcare | 154 | 25.9% | 440 | 74.1% |
| **Region** | Northern Italy | 454 | 35.7% | 819 | 64.3% |
|  | Centre Italy | 85 | 37.3% | 143 | 62.7% |
|  | Southern Italy plus islands | 126 | 35.8% | 226 | 64.2% |
|  | Foreign countries | 8 | 38.1% | 13 | 61.9% |
| **Blood donor** | Yes | 125 | 38.8% | 197 | 61.2% |
|  | In the past | 101 | 37.1% | 171 | 62.9% |
|  | No | 447 | 34.9% | 833 | 65.1% |
| **Post mortem organs donation** | Yes | 527 | 34.9% | 982 | 65.1% |
|  | No/don't know/I prefer do not answer | 146 | 40.0% | 219 | 60.0% |
| **Relatives and friends suffering from rare diseases with no cure** | No | 489 | 36.0% | 869 | 64.0% |
|  | Yes. I have had experience with my loved ones | 148 | 37.4% | 248 | 62.6% |
|  | Yes. I have had personal experience | 36 | 30.0% | 84 | 70.0% |
| **Religion*** | Not religious/I prefer do not answer | 249 | 31.9% | 532 | 68.1% |
|  | Religious | 424 | 38.8% | 669 | 61.2% |
| **Rate of information*** | Daily | 421 | 34.2% | 811 | 65.8% |
|  | At least once per week | 192 | 36.6% | 332 | 63.4% |
|  | Once per month or less | 60 | 50.8% | 58 | 49.2% |
| **Sources of information about news** | Online newspaper | 312 | 34.5% | 593 | 65.5% |
|  | Printed newspaper | 30 | 41.7% | 42 | 58.3% |
|  | Radio | 38 | 35.2% | 70 | 64.8% |
|  | TV | 165 | 36.4% | 288 | 63.6% |
|  | Social network | 74 | 44.0% | 94 | 56.0% |
|  | Other | 54 | 32.1% | 114 | 67.9% |

*Row percentages*

**p-value≤0.05. **p-value≤0.001*

**Supplementary Table 2. Individuals’ characteristics of the study participants by response to question 4 “I would donate a blood sample for the generation of hiPSCs to treat only my relatives, close friends or me“ (n=1201, 64.1%)**

|  | | **I disagree (n=1033, 86.0%)** | | **Don't know (n=63, 5.2%)** | | **I agree (n=105, 8.7%)** | |
| --- | --- | --- | --- | --- | --- | --- | --- |
|  |  | **N** | **%** | **N** | **%** | **N** | **%** |
| **Gender** | Men | 351 | 85.0% | 22 | 5.3% | 40 | 9.7% |
|  | Women | 679 | 86.7% | 40 | 5.1% | 64 | 8.2% |
|  | Other | 3 | 60.0% | 1 | 20.0% | 1 | 20.0% |
| **Age*** | 18-35 | 432 | 88.9% | 26 | 5.3% | 28 | 5.8% |
|  | 36-55 | 351 | 85.0% | 22 | 5.3% | 40 | 9.7% |
|  | ≥56 | 250 | 82.8% | 15 | 5.0% | 37 | 12.3% |
| **Education**** | Undergraduated | 279 | 80.4% | 19 | 5.5% | 49 | 14.1% |
|  | Graduated | 754 | 88.3% | 44 | 5.2% | 56 | 6.6% |
| **Marital status*** | Married | 446 | 82.9% | 27 | 5.0% | 65 | 12.1% |
|  | Civil union | 102 | 86.4% | 8 | 6.8% | 8 | 6.8% |
|  | Single | 365 | 89.2% | 21 | 5.1% | 23 | 5.6% |
|  | Separated/divorced | 70 | 88.6% | 4 | 5.1% | 5 | 6.3% |
|  | Widow/er | 14 | 82.4% | 0 | 0.0% | 3 | 17.6% |
|  | I prefer do not answer | 36 | 90.0% | 3 | 7.5% | 1 | 2.5% |
| **Occupation*** | Worker with permanent position | 473 | 85.2% | 30 | 5.4% | 52 | 9.4% |
|  | Self-employed | 154 | 82.4% | 16 | 8.6% | 17 | 9.1% |
|  | Worker with temporary position | 125 | 86.8% | 9 | 6.3% | 10 | 6.9% |
|  | Unemployed | 29 | 90.6% | 0 | 0.0% | 3 | 9.4% |
|  | Housewife | 18 | 69.2% | 1 | 3.8% | 7 | 26.9% |
|  | Retired | 71 | 86.6% | 2 | 2.4% | 9 | 11.0% |
|  | Student | 163 | 93.1% | 5 | 2.9% | 7 | 4.0% |
| **Job area** | Other | 651 | 85.5% | 41 | 5.4% | 69 | 9.1% |
|  | Healthcare | 382 | 86.8% | 22 | 5.0% | 36 | 8.2% |
| **Region** | Northern Italy | 715 | 87.3% | 42 | 5.1% | 62 | 7.6% |
|  | Centre Italy | 123 | 86.0% | 7 | 4.9% | 13 | 9.1% |
|  | Southern Italy plus islands | 184 | 81.4% | 14 | 6.2% | 28 | 12.4% |
|  | Foreign countries | 11 | 84.6% | 0 | 0.0% | 2 | 15.4% |
| **Blood donor** | Yes | 175 | 88.8% | 9 | 4.6% | 13 | 6.6% |
|  | In the past | 147 | 86.0% | 10 | 5.8% | 14 | 8.2% |
|  | No | 711 | 85.4% | 44 | 5.3% | 78 | 9.4% |
| **Post mortem organs donation**** | Yes | 871 | 88.7% | 39 | 4.0% | 72 | 7.3% |
|  | No/don't know/I prefer do not answer | 162 | 74.0% | 24 | 11.0% | 33 | 15.1% |
| **Relatives and friends suffering from rare diseases with no cure*** | No | 743 | 85.5% | 55 | 6.3% | 71 | 8.2% |
|  | Yes. I have had experience with my loved ones | 220 | 88.7% | 8 | 3.2% | 20 | 8.1% |
|  | Yes. I have had personal experience | 70 | 83.3% | 0 | 0.0% | 14 | 16.7% |
| **Religion**** | Not religious/I prefer do not answer | 481 | 90.4% | 22 | 4.1% | 29 | 5.5% |
|  | Religious | 552 | 82.5% | 41 | 6.1% | 76 | 11.4% |
| **Rate of information*** | Daily | 699 | 86.2% | 40 | 4.9% | 72 | 8.9% |
|  | At least once per week | 287 | 86.4% | 19 | 5.7% | 26 | 7.8% |
|  | Once per month or less | 47 | 81.0% | 4 | 6.9% | 7 | 12.1% |
| **Sources of information about news*** | Online newspaper | 524 | 88.4% | 28 | 4.7% | 41 | 6.9% |
|  | Printed newspaper | 38 | 90.5% | 2 | 4.8% | 2 | 4.8% |
|  | Radio | 67 | 95.7% | 2 | 2.9% | 1 | 1.4% |
|  | TV | 236 | 81.9% | 15 | 5.2% | 37 | 12.8% |
|  | Social network | 74 | 78.7% | 9 | 9.6% | 11 | 11.7% |
|  | Other | 94 | 82.5% | 7 | 6.1% | 13 | 11.4% |

*Row percentages*

**p-value≤0.05. **p-value≤0.001*

**Supplementary Table 3. Individuals’ characteristics of the study participants by response to question 6 “I would accept that hiPSCs obtained from my blood sample would be used in experiments on animals” (n=1201. 64.1%)**

|  | | **I disagree (n=305, 25.4%)** | | **Don't know (n=242, 20.1%)** | | **I agree (n=654, 54.5%)** | |
| --- | --- | --- | --- | --- | --- | --- | --- |
|  |  | **N** | **%** | **N** | **%** | **N** | **%** |
| **Gender**** | Men | 71 | 17.2% | 84 | 20.3% | 258 | 62.5% |
|  | Women | 232 | 29.6% | 157 | 20.1% | 394 | 50.3% |
|  | Other | 2 | 40.0% | 1 | 20.0% | 2 | 40.0% |
| **Age**** | 18-35 | 89 | 18.3% | 97 | 20.0% | 300 | 61.7% |
|  | 36-55 | 130 | 31.5% | 90 | 21.8% | 193 | 46.7% |
|  | ≥56 | 86 | 28.5% | 55 | 18.2% | 161 | 53.3% |
| **Education**** | Undergraduated | 113 | 32.6% | 82 | 23.6% | 152 | 43.8% |
|  | Graduated | 192 | 22.5% | 160 | 18.7% | 502 | 58.8% |
| **Marital status*** | Married | 144 | 26.8% | 108 | 20.1% | 286 | 53.2% |
|  | Civil union | 34 | 28.8% | 24 | 20.3% | 60 | 50.8% |
|  | Single | 78 | 19.1% | 88 | 21.5% | 243 | 59.4% |
|  | Separated/divorced | 30 | 38.0% | 14 | 17.7% | 35 | 44.3% |
|  | Widow/er | 7 | 41.2% | 3 | 17.6% | 7 | 41.2% |
|  | I prefer do not answer | 12 | 30.0% | 5 | 12.5% | 23 | 57.5% |
| **Occupation**** | Worker with permanent position | 152 | 27.4% | 111 | 20.0% | 292 | 52.6% |
|  | Self-employed | 61 | 32.6% | 48 | 25.7% | 78 | 41.7% |
|  | Worker with temporary position | 25 | 17.4% | 26 | 18.1% | 93 | 64.6% |
|  | Unemployed | 14 | 43.8% | 7 | 21.9% | 11 | 34.4% |
|  | Housewife | 11 | 42.3% | 5 | 19.2% | 10 | 38.5% |
|  | Retired | 20 | 24.4% | 14 | 17.1% | 48 | 58.5% |
|  | Student | 22 | 12.6% | 31 | 17.7% | 122 | 69.7% |
| **Job area**** | Other | 218 | 28.6% | 183 | 24.0% | 360 | 47.3% |
|  | Healthcare | 87 | 19.8% | 59 | 13.4% | 294 | 66.8% |
| **Region** | Northern Italy | 197 | 24.1% | 161 | 19.7% | 461 | 56.3% |
|  | Centre Italy | 45 | 31.5% | 29 | 20.3% | 69 | 48.3% |
|  | Southern Italy plus islands | 57 | 25.2% | 51 | 22.6% | 118 | 52.2% |
|  | Foreign countries | 6 | 46.2% | 1 | 7.7% | 6 | 46.2% |
| **Blood donor** | Yes | 47 | 23.9% | 28 | 14.2% | 122 | 61.9% |
|  | In the past | 43 | 25.1% | 34 | 19.9% | 94 | 55.0% |
|  | No | 215 | 25.8% | 180 | 21.6% | 438 | 52.6% |
| **Post mortem organs donation**** | Yes | 229 | 23.3% | 198 | 20.2% | 555 | 56.5% |
|  | No/don't know/I prefer do not answer | 76 | 34.7% | 44 | 20.1% | 99 | 45.2% |
| **Relatives and friends suffering from rare diseases with no cure** | No | 228 | 26.2% | 178 | 20.5% | 463 | 53.3% |
|  | Yes. I have had experience with my loved ones | 53 | 21.4% | 51 | 20.6% | 144 | 58.1% |
|  | Yes. I have had personal experience | 24 | 28.6% | 13 | 15.5% | 47 | 56.0% |
| **Religion** | Not religious/I prefer do not answer | 127 | 23.9% | 109 | 20.5% | 296 | 55.6% |
|  | Religious | 178 | 26.6% | 133 | 19.9% | 358 | 53.5% |
| **Rate of information** | Daily | 213 | 26.3% | 160 | 19.7% | 438 | 54.0% |
|  | At least once per week | 75 | 22.6% | 69 | 20.8% | 188 | 56.6% |
|  | Once per month or less | 17 | 29.3% | 13 | 22.4% | 28 | 48.3% |
| **Sources of information about news** | Online newspaper | 128 | 21.6% | 130 | 21.9% | 335 | 56.5% |
|  | Printed newspaper | 12 | 28.6% | 7 | 16.7% | 23 | 54.8% |
|  | Radio | 24 | 34.3% | 11 | 15.7% | 35 | 50.0% |
|  | TV | 80 | 27.8% | 60 | 20.8% | 148 | 51.4% |
|  | Social network | 29 | 30.9% | 15 | 16.0% | 50 | 53.2% |
|  | Other | 32 | 28.1% | 19 | 16.7% | 63 | 55.3% |

*Row percentages*

**p-value≤0.05. **p-value≤0.001*

**Supplementary Table 4. Individuals’ characteristics of the study participants by response to question 7 “I am concerned about the current research and therapy applications of these new stem cells” (n=1201. 64.1%)**

|  | | **I disagree (n=857, 71.4%)** | | **Don't know (n=261, 21.7%)** | | **I agree (n=83, 6.9%)** | |
| --- | --- | --- | --- | --- | --- | --- | --- |
|  |  | **N** | **%** | **N** | **%** | **N** | **%** |
| **Gender*** | Men | 313 | 75.8% | 71 | 17.2% | 29 | 7.0% |
|  | Women | 542 | 69.2% | 188 | 24.0% | 53 | 6.8% |
|  | Other | 2 | 40.0% | 2 | 40.0% | 1 | 20.0% |
| **Age**** | 18-35 | 382 | 78.6% | 87 | 17.9% | 17 | 3.5% |
|  | 36-55 | 268 | 64.9% | 111 | 26.9% | 34 | 8.2% |
|  | ≥56 | 207 | 68.5% | 63 | 20.9% | 32 | 10.6% |
| **Education**** | Undergraduated | 210 | 60.5% | 108 | 31.1% | 29 | 8.4% |
|  | Graduated | 647 | 75.8% | 153 | 17.9% | 54 | 6.3% |
| **Marital status*** | Married | 373 | 69.3% | 114 | 21.2% | 51 | 9.5% |
|  | Civil union | 88 | 74.6% | 26 | 22.0% | 4 | 3.4% |
|  | Single | 305 | 74.6% | 87 | 21.3% | 17 | 4.2% |
|  | Separated/divorced | 52 | 65.8% | 19 | 24.1% | 8 | 10.1% |
|  | Widow/er | 9 | 52.9% | 6 | 35.3% | 2 | 11.8% |
|  | I prefer do not answer | 30 | 75.0% | 9 | 22.5% | 1 | 2.5% |
| **Occupation**** | Worker with permanent position | 368 | 66.3% | 141 | 25.4% | 46 | 8.3% |
|  | Self-employed | 134 | 71.7% | 41 | 21.9% | 12 | 6.4% |
|  | Worker with temporary position | 118 | 81.9% | 17 | 11.8% | 9 | 6.3% |
|  | Unemployed | 21 | 65.6% | 10 | 31.3% | 1 | 3.1% |
|  | Housewife | 14 | 53.8% | 9 | 34.6% | 3 | 11.5% |
|  | Retired | 60 | 73.2% | 15 | 18.3% | 7 | 8.5% |
|  | Student | 142 | 81.1% | 28 | 16.0% | 5 | 2.9% |
| **Job area**** | Other | 495 | 65.0% | 210 | 27.6% | 56 | 7.4% |
|  | Healthcare | 362 | 82.3% | 51 | 11.6% | 27 | 6.1% |
| **Region** | Northern Italy | 583 | 71.2% | 173 | 21.1% | 63 | 7.7% |
|  | Centre Italy | 97 | 67.8% | 39 | 27.3% | 7 | 4.9% |
|  | Southern Italy plus islands | 169 | 74.8% | 44 | 19.5% | 13 | 5.8% |
|  | Foreign countries | 8 | 61.5% | 5 | 38.5% | 0 | 0.0% |
| **Blood donor** | Yes | 143 | 72.6% | 39 | 19.8% | 15 | 7.6% |
|  | In the past | 120 | 70.2% | 39 | 22.8% | 12 | 7.0% |
|  | No | 594 | 71.3% | 183 | 22.0% | 56 | 6.7% |
| **Post mortem organs donation**** | Yes | 728 | 74.1% | 192 | 19.6% | 62 | 6.3% |
|  | No/don't know/I prefer do not answer | 129 | 58.9% | 69 | 31.5% | 21 | 9.6% |
| **Relatives and friends suffering from rare diseases with no cure** | No | 617 | 71.0% | 198 | 22.8% | 54 | 6.2% |
|  | Yes. I have had experience with my loved ones | 178 | 71.8% | 47 | 19.0% | 23 | 9.3% |
|  | Yes. I have had personal experience | 62 | 73.8% | 16 | 19.0% | 6 | 7.1% |
| **Religion**** | Not religious/I prefer do not answer | 404 | 75.9% | 108 | 20.3% | 20 | 3.8% |
|  | Religious | 453 | 67.7% | 153 | 22.9% | 63 | 9.4% |
| **Rate of information** | Daily | 588 | 72.5% | 166 | 20.5% | 57 | 7.0% |
|  | At least once per week | 235 | 70.8% | 74 | 22.3% | 23 | 6.9% |
|  | Once per month or less | 34 | 58.6% | 21 | 36.2% | 3 | 5.2% |
| **Sources of information about news** | Online newspaper | 432 | 72.8% | 114 | 19.2% | 47 | 7.9% |
|  | Printed newspaper | 31 | 73.8% | 6 | 14.3% | 5 | 11.9% |
|  | Radio | 43 | 61.4% | 22 | 31.4% | 5 | 7.1% |
|  | TV | 195 | 67.7% | 77 | 26.7% | 16 | 5.6% |
|  | Social network | 68 | 72.3% | 21 | 22.3% | 5 | 5.3% |
|  | Other | 88 | 77.2% | 21 | 18.4% | 5 | 4.4% |

*Row percentages*

**p-value≤0.05. **p-value≤0.001*

**Supplementary Table 5. Individuals’ characteristics of the study participants by response to question 8 “I am concerned about the management of my personal data in relation to storage and use of the new stem cells derived from my blood cells” (n=1201. 64.1%)**

|  | | **I disagree (n=779, 64.9%)** | | **Don't know (n=206, 17.2%)** | | **I agree (n=216, 18.0%)** | |
| --- | --- | --- | --- | --- | --- | --- | --- |
|  |  | **N** | **%** | **N** | **%** | **N** | **%** |
| **Gender** | Men | 264 | 63.9% | 63 | 15.3% | 86 | 20.8% |
|  | Women | 513 | 65.5% | 142 | 18.1% | 128 | 16.3% |
|  | Other | 2 | 40.0% | 1 | 20.0% | 2 | 40.0% |
| **Age**** | 18-35 | 357 | 73.5% | 75 | 15.4% | 54 | 11.1% |
|  | 36-55 | 254 | 61.5% | 70 | 16.9% | 89 | 21.5% |
|  | ≥56 | 168 | 55.6% | 61 | 20.2% | 73 | 24.2% |
| **Education*** | Undergraduated | 210 | 60.5% | 76 | 21.9% | 61 | 17.6% |
|  | Graduated | 569 | 66.6% | 130 | 15.2% | 155 | 18.1% |
| **Marital status*** | Married | 326 | 60.6% | 100 | 18.6% | 112 | 20.8% |
|  | Civil union | 82 | 69.5% | 12 | 10.2% | 24 | 20.3% |
|  | Single | 288 | 70.4% | 69 | 16.9% | 52 | 12.7% |
|  | Separated/divorced | 50 | 63.3% | 13 | 16.5% | 16 | 20.3% |
|  | Widow/er | 9 | 52.9% | 5 | 29.4% | 3 | 17.6% |
|  | I prefer do not answer | 24 | 60.0% | 7 | 17.5% | 9 | 22.5% |
| **Occupation*** | Worker with permanent position | 344 | 62.0% | 98 | 17.7% | 113 | 20.4% |
|  | Self-employed | 117 | 62.6% | 33 | 17.6% | 37 | 19.8% |
|  | Worker with temporary position | 107 | 74.3% | 16 | 11.1% | 21 | 14.6% |
|  | Unemployed | 20 | 62.5% | 8 | 25.0% | 4 | 12.5% |
|  | Housewife | 11 | 42.3% | 7 | 26.9% | 8 | 30.8% |
|  | Retired | 48 | 58.5% | 16 | 19.5% | 18 | 22.0% |
|  | Student | 132 | 75.4% | 28 | 16.0% | 15 | 8.6% |
| **Job area*** | Other | 472 | 62.0% | 146 | 19.2% | 143 | 18.8% |
|  | Healthcare | 307 | 69.8% | 60 | 13.6% | 73 | 16.6% |
| **Region** | Northern Italy | 534 | 65.2% | 133 | 16.2% | 152 | 18.6% |
|  | Centre Italy | 94 | 65.7% | 27 | 18.9% | 22 | 15.4% |
|  | Southern Italy plus islands | 142 | 62.8% | 43 | 19.0% | 41 | 18.1% |
|  | Foreign countries | 9 | 69.2% | 3 | 23.1% | 1 | 7.7% |
| **Blood donor** | Yes | 138 | 70.1% | 29 | 14.7% | 30 | 15.2% |
|  | In the past | 111 | 64.9% | 29 | 17.0% | 31 | 18.1% |
|  | No | 530 | 63.6% | 148 | 17.8% | 155 | 18.6% |
| **Post mortem organs donation**** | Yes | 664 | 67.6% | 160 | 16.3% | 158 | 16.1% |
|  | No/don't know/I prefer do not answer | 115 | 52.5% | 46 | 21.0% | 58 | 26.5% |
| **Relatives and friends suffering from rare diseases with no cure** | No | 557 | 64.1% | 161 | 18.5% | 151 | 17.4% |
|  | Yes. I have had experience with my loved ones | 166 | 66.9% | 35 | 14.1% | 47 | 19.0% |
|  | Yes. I have had personal experience | 56 | 66.7% | 10 | 11.9% | 18 | 21.4% |
| **Religion*** | Not religious/I prefer do not answer | 370 | 69.5% | 85 | 16.0% | 77 | 14.5% |
|  | Religious | 409 | 61.1% | 121 | 18.1% | 139 | 20.8% |
| **Rate of information** | Daily | 524 | 64.6% | 133 | 16.4% | 154 | 19.0% |
|  | At least once per week | 224 | 67.5% | 60 | 18.1% | 48 | 14.5% |
|  | Once per month or less | 31 | 53.4% | 13 | 22.4% | 14 | 24.1% |
| **Sources of information about news*** | Online newspaper | 404 | 68.1% | 84 | 14.2% | 105 | 17.7% |
|  | Printed newspaper | 29 | 69.0% | 5 | 11.9% | 8 | 19.0% |
|  | Radio | 39 | 55.7% | 11 | 15.7% | 20 | 28.6% |
|  | TV | 175 | 60.8% | 66 | 22.9% | 47 | 16.3% |
|  | Social network | 65 | 69.1% | 16 | 17.0% | 13 | 13.8% |
|  | Other | 67 | 58.8% | 24 | 21.1% | 23 | 20.2% |

*Row percentages*

**p-value≤0.05. **p-value≤0.001*
